# Lymph node infarction – a rare complication associated with disseminated intra vascular coagulation in a case of dengue fever

**DOI:** 10.1186/1472-6890-5-11

**Published:** 2005-12-12

**Authors:** I Satish Rao, Anand C Loya, KS Ratnakar, VR Srinivasan

**Affiliations:** 1 Department of Pathology, Nizam's Institute of Medical Sciences, Hyderabad, India; 2 Department of General Medicine, Nizam's Institute of Medical Sciences, Hyderabad, India

## Abstract

**Background:**

Lymph node infarction is known to occur in association with many non-neoplastic and neoplastic conditions however its occurrence in association with DIC is not reported hitherto in the literature.

**Case presentation:**

We describe an unusual case of lymph node infarction in a twenty seven year old male following disseminated intravascular coagulation (DIC) in a case of dengue fever. Multiple sections of the infarcted and the surrounding non-infarcted lymph nodes failed to reveal any predisposing condition. How ever the parahilar vessels showed thrombotic occlusion, which must have been responsible for the infarction.

**Conclusion:**

Global infarction of the lymph node may mask the underlying pathology. Any malignancy especially lymphoma may coexist or follow lymph node infarction, therefore the patient needs constant surveillance.

## Background

Lymph node infarction is a rare occurrence as it has abundant vascularity. Infarction can be due to various non-neoplastic and neoplastic conditions. It may frequently fore shadow neoplastic conditions especially lymphoma and a diligent search should be undertaken to identify the cause. We present a case of lymph node infarction detected in association with disseminated intravascular coagulation in a serologically proven case of Dengue fever.

## Case presentation

This twenty-seven years old male presented with complaints of fever with rigor and chills and sore throat for ten days. It was associated with myalgia and recurrent vomiting followed by diffuse maculopapular rash all over the body with sparing of mucous membranes and periorbital tissues. On examination, he had conjunctival congestion with toxic facies. He had a past history of gout for which he received allolpurinol for twenty days and phenytoin for sudden loss of consciousness in another institution. There was no past history of tuberculosis, diabetes or hypertension.

Bilateral hard indurated cervical lymphadenopathy and bilateral inguinal lymphadenopathy was also observed along with bilateral parotid and submandibular gland enlargement. Hepatosplenomegaly was detected on ultrasonography. Hemogram on several occasions from admission revealed normal hemoglobin and red blood cell morphology. There was transient neutrophilic leukocytosis, whereas platelet counts and erythrocyte sedimentation rate were normal throughout. During his treatment with ceftriaxone, he suddenly developed shock, cyanosis and acute renal failure for which he was given inotropic support, amoxicillin, ceftazidine and steroids. He gradually responded and recovered in due course of time. A clinical diagnosis of shock syndrome due to Dengue fever or Epstein Barr virus (EBV) or streptococcus or phenytoin induced hypersensitivity syndrome was made. The Dengue virus IgM antibody Elisa was positive and serum D-dimer levels were positive (>200 ng/ml). Anti EBV IgG antibody test was positive but EBV IgM antibody test was negative. The serology tests for anti- cytomegalovirus antibody, anti leptospira antibody, Widal test, brucella (abortus and melitensis), and HIV were negative. The blood, throat swab and urine cultures were sterile. Paul-Bunnel and Weil-Felix tests were negative. Cervical lymph node biopsy was undertaken for histopathology.

**Figure 1:**
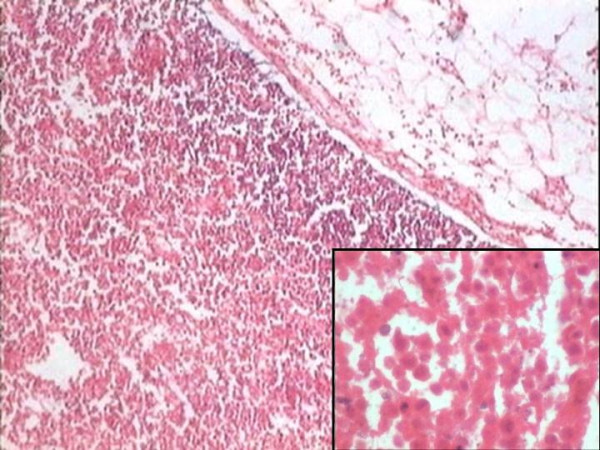
Lymph node infarction with viable rim of sub capsular lymphoid tissue. (40X, H & E) Inset: Note the cellular outlines of the individual lymphocytes, which have undergone coagulative necrosis. (400X, H&E).

**Figure 2:**
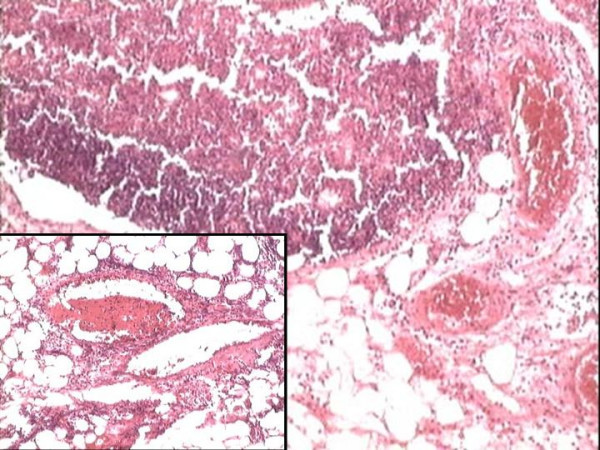
Perinodal blood vessels show marked congestion (H&E-40X), and fibrin thrombi (Inset-H&E-100X).

Gross examination revealed four lymph nodes, the largest one measuring 1.5 cm and the rest below 1 cm. On microscopy, two of the lymph nodes (including the largest one) showed near total infarction of the parenchyma with preservation of cellular outlines of the lymphocytes (Fig 1). A thin rim of viable subcapsular normal mature lymphocytes along with neutrophils and plasma cells were identified. The medullary vessels showed vascular congestion however the blood vessels in the perinodal adipose tissue showed multiple fibrin thrombi with congestion in some of the vessels (Fig 2).

**Figure 3:**
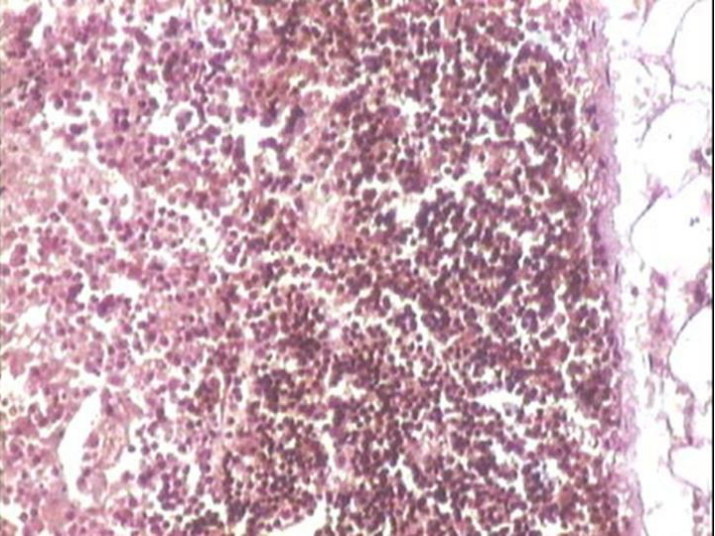
CD20 immunohistochemistry highlights the viable subcapsular lymphoid tissue, where as the adjacent infarcted area also shows focal positivity (H&E-200X).

Multiple deeper sections revealed perinodal granulation tissue with viable subcapsular lymphoid tissue but no atypical cells, histiocytes or granulomas were seen. Stains for fungi and acid-fast bacilli were negative. The adjacent two uninvolved lymph nodes showed features of reactive change. Immunohistochemistry with B- cell (CD20) and T- cell (CD3) markers was performed. The viable lymphoid tissue showed an admixture of both B and T-cells. The infarcted tissue showed variable positivity and these markers highlighted few reactive follicles (Fig 3). The uninvolved lymph nodes also showed a normal positivity pattern.

The patient was asymptomatic thereafter and had complete regression of the lymph nodes. He was lost to follow up after two months of discharge from the hospital.

Lymph node infarction is a rare phenomenon reported in literature in association with various neoplastic and non-neoplastic conditions. The non-neoplastic conditions associated with infarction are namely polyarteritis nodosa, viral infections (Parvovirus B19, Infectious mononucleosis), cholesterol atheromatous embolism, thrombosis, gold injections, intestinal volvulus, postmediastinoscopy, mononeuritis multiplex, fine needle aspiration cytology and finally may be idiopathic [1-11]. Hemorrhagic infarction of hilar lymph nodes has also been reported recently in association with heart lung transplantation. [1,2]

The neoplastic lesions most commonly associated with infarction are malignant lymphoma and metastatic melanoma [1,13-15]. The differential diagnosis pertaining to the present case would be the above mentioned two conditions along with necrotizing lymphadenitis (Kikuchi's lymphadenitis), mucocutaneous lymph node syndrome, and necrotizing granulomatous inflammation.

Serial deeper sections failed to reveal any evidence of either lymphoma or melanoma, any granulomatous inflammation, and vasculitis or monocytoid histiocytes, thus ruling out the above mentioned conditions. Drug induced hypersensitivity reactions in a lymph node especially due to phenytoin are known in the form of inter follicular expansion by a mixed infiltrate of immunoblasts, plasma cells and eosinophils with varying degrees of vascular proliferation. Focal necrosis has also been observed. Other drugs have been reported to show reactive change [16]. These changes were not seen in the present case in the non-infarcted lymph nodes. In the present case it appears that infarction of the lymph node occurred before the DIC became clinically evident.

A completely infarcted lymph node should alert the pathologist to the high possibility of malignant lymphoma, as it is the most common cause of spontaneous lymph node infarction [5]. Further investigation can be carried out by taking multiple deeper sections of the infarcted lymph node to visualize the viable subcapsular tissue for any clue of malignancy. Immunohistochemistry has been done on the infarcted lymph node sections to establish the underlying pathology. Although it is widely believed that necrotic tissue is not suitable for immunohistochemical study, this view may be inaccurate. [17-20]

The detection of spontaneous lymph node infarction should alert the pathologist and the clinician alike for an associated or underlying impending pathology. In a multicentre study of 51 cases of lymph-node infarction seen in the 30-year period, Maurer et al identified 14 cases of malignant lymphoma synchronously with the infarct. Of the remaining 37 patients, six showed manifestations of malignant lymphoma in the follow-up period within 2 years of the lymph-node infarction.

On analyzing other cases in the literature these authors also confirmed that a minority (26 of 81) have developed malignant lymphoma, which appeared within 2 years of infarction. Therefore, a thorough examination of both the infarcted lymph nodes and others resected at the same time is mandatory in order to exclude concomitant or underlying malignant lymphoma. The risk of development of malignant lymphoma after infarction was found to be negligible after two years [15].

## Conclusion

We report a very unusual case of lymph node infarction associated with disseminated intra-vascular coagulation in dengue fever not yet reported in the literature. The simple reason may be that usually lymph nodes are not sampled in DIC. However, any infarcted lymph node should be eyed with suspicion especially when it is enlarged in size. Multiple deeper sections and immunohistochemistry may be very useful to unearth the underlying cause and finally when the cause is not found out, these patients should be followed up for a minimum of 2 years.

## List of abbreviations

**1) **DIC – Disseminated intravascular coagulation

**2) **ESR – Erythrocyte sedimentation rate

**3) **EBV – Epstein Barr virus

**4) **IgM – Immunoglobulin M

**5) **IgG – Immunoglobulin G

**6) **HIV – Human immunodeficiency virus

## Competing interests

The author(s) declare that they have no competing interests.

## Authors' contributions

ISR – Has made substantial contribution to conception, design, data analysis & interpretation. Has drafted the article and finally approved the article for publication.

ACL – Has made significant contribution in conception, design and co-ordination. Also helped in drafting and revising critically the content of the article.

KSR – Has made critical analysis of the article and approved the article for submission.

VRS – Has provided clinical details and analyzed the article.

## Pre-publication history

The pre-publication history for this paper can be accessed here:


